# Peer Review of “Offenders With Personality Disorder Who Fail to Progress: A Case-Control Study Using Partial Least Squares Structural Equation Modeling Path Analysis”

**DOI:** 10.2196/33936

**Published:** 2021-10-29

**Authors:** Martina Sonnweber

**Affiliations:** 1 Department of Forensic Psychiatry Psychiatric Hospital University of Zurich Rheinau Switzerland

*This is a peer-review report submitted for the paper “Offenders With Personality Disorder Who Fail to Progress: A Case-Control Study Using Partial Least Squares Structural Equation Modeling Path Analysis”.*

## Round 1 Review

### General Comments

Thank you for the invitation to review “Offenders with Personality Disorder who Fail to Progress: A Case Control Study Using PLS-SEM Path Analysis”. The paper [1] aims at identifying and describing a subgroup of offenders with personality disorders (PDs) that fails to progress in treatment. The method of choice is a structural equation model and allows for modeling and analyzing causal paths of several latent variables and complex interrelations of a range of variables. Outcomes are consistent with the literature that has identified factors associated with nonparticipation in treatment and include a risk assessed as inaccessible to intervention; negative attitudes toward treatment, such as low motivation; and psychopathology. Low treatment motivation was also found to predict problematic institutional behavior. Strengths and limitations are presented and include the critical assessment of the methodological approach and the problem of missing data.

I liked reading your paper and think that it is of great importance to analyze certain subgroups in more detail and with statistical approaches that incorporate latent variables and unravel causal structures.

However, I do not like the fact that many different models are mentioned in the Introduction and that these models are not mentioned further on. These parts could be shortened, and the paper would certainly benefit from this. I found the subdivision of the Introduction into many subunits disruptive as well. There is no common thread to the Introduction. Please try to create a better text flow.

I did like the Methods section and the Discussion, which was balanced and informative for the most part.

### Specific Comments

#### Major Comments

1. A distinction between the different PDs would be appropriate, as the risk for offending is not the same for all PDs.
2. Additionally, please give some more information about PDs in general.
3. “The OPD [Offenders Personality Disorder] pathway is informed from the “What Works?” literature [2], the RNR [risk needs responsivity] principles [3] and the Good Lives Model (GLM; [4]. However, the RNR model been criticised for not providing clear guidance for therapists for engaging offenders lacking in treatment readiness [5]. The responsivity principle of the RNR model may not currently be effectively implemented in the OPD pathway and contributing to the problem of offenders being referred but not accepted to numerous OPD services.” Incorporating these resources adds no value in my opinion, since there is no further information about these models.
4. Attitudes towards treatment”: Please specify possible outcomes in the description.
5. A descriptive visual representation of the analysis plan would be helpful.
6. Null hypothesis significance testing (NHST) needs hypotheses. If you use NHST (ie, *χ*^2^ and t test) in part 1 of the analysis, you need to formulate hypotheses since blind testing always leads to results.
7. No correction (eg, Bonferroni) was made, despite multiple testing. This should be done. If one does not do this, it is fine because the main goal of the study was the model, but this needs to be addressed and reflected upon.
8. Methodology and results flow into each other. Please find a way to separate this better. I would also like to see not only the inner model described in the methodology but also the outer model (eg, the factors of psychopathology).
9. The latent variables create the inner model and the variables were connected using clinical knowledge and theory (Figure 1).”
  Please specify “clinical knowledge” and “theory.” This is quite speculative. In addition, Figure 1 would benefit from a more specific description.
10. “Second, the outer loadings within the SEM [structural equation modeling] model suggest that the single most influential factor was psychopathy or psychopathic disorder, which has long been acknowledged as a limiting factor for treatment and rehabilitation [6]. It could be argued that psychopathic offenders are not best served on a pathway that caters for offenders with personality disorder in the broader sense of the diagnosis as their needs are known to be different [7].”
  Do you have a suggestion for these individuals?
11. Other limitations are that you specify a model a priori and that so many factors are used for such a complex phenomenon (with a quite limited sample).
12. In addition, one must see very critically that psychosis and PS are combined. The fact must be discussed as this is problematic, having a massive influence on therapy and behavior.
13. Please consider dividing the Conclusions section into Future Perspectives and Conclusions sections as this seems kind of inconsistent.

#### Minor Comments

1. Please consider changing the title of the paper by omitting “PLS-SEM [partial least squares structural equation modeling] Path Analysis,” which is too technical in my opinion (or maybe do not use the abbreviation).
2. Please divide the sentence in the results section of the Abstract into two sentences and thereby avoiding the semicolon.
3. Please avoid the semicolons in the second paragraph and check overall structure of the sentences (and use hyphens if appropriate). Check for missed words, sentence structure, and punctuation in the paper.
4. Stay consistent when using abbreviations—do not alternate “PD” and “personality disorder”.
5. Please define “NHS” (National Health Service) before using the abbreviation.
6. I would like to read more about the “screening algorithm” and which PDs this algorithm screens for.
7. “However, several of us are clinicians working within the London Pathways Partnership (LPP), a consortium of NHS trusts delivering services within the OPD pathway, are aware of several individuals that no OPD service, in prison or the NHS, is prepared to accept.” Reformulate this sentence since it is not comprehensible easily.
8. Omit % in the brackets in each row of the tables.
9. Although the relationship between problematic custodial behaviour and service refusal was not strong, the results still emphasise that services aiming to support these individuals need be able to receive men with patterns of such behaviour and contain and manage ongoing episodes, without this resulting in treatment termination.” Please rephrase this, as it is a quite complicated sentence.

## Abbreviations

GLM: Good Lives Model
LPP: London Pathways Partnership
NHS: National Health Service
NHST: null hypothesis significance testing
OPD: Offenders Personality Disorder
PD: personality disorder
PLS-SEM: partial least squares structural equation modeling
RNR: risk needs responsivity
SEM: structural equation modeling
